# Unilateral Vision Loss in Elderly People in Residential Care: Prevalence, Causes and Impact on Visual Functioning: The Hyderabad Ocular Morbidity in Elderly Study (HOMES)

**DOI:** 10.1080/09286586.2022.2104323

**Published:** 2022-07-27

**Authors:** Srinivas Marmamula, Navya Rekha Barrenkala, Thirupathi Reddy Kumbham, Satya Brahmanandam Modepalli, Jill Keeffe

**Affiliations:** a Allen Foster Community Eye Health Research Centre, Gullapalli Pratibha Rao International Centre for Advancement of Rural Eye care, L V Prasad Eye Institute, Hyderabad, India; b Brien Holden Institute of Optometry and Vision Science, L V Prasad Eye Institute, Hyderabad, India;; c Department of Biotechnology/Wellcome Trust India Alliance, L V Prasad Eye Institute, Hyderabad, India;; d School of Optometry and Vision Science, University of New South Wales, Sydney, Australia

**Keywords:** Unilateral visual impairment, elderly, residential care, India, HOMES, visual function

## Abstract

**Purpose:**

To investigate the prevalence, causes and impact of unilateral visual impairment (UVI) on visual function in the elderly in ‘home for the aged’ in Hyderabad, India.

**Methods:**

Participants aged ≥60 years were recruited from 41 ‘homes for the aged’. All participants had complete eye examinations including visual acuity assessment, refraction, slit-lamp and fundus examination. Unilateral visual impairment (UVI) was defined as presenting VA worse than 6/18 in one eye and presenting VA 6/18 or better in the other eye. Indian Vision Function Questionnaire (INDVFQ) was used for assessing visual functioning.

**Results:**

Of the total 1,513 elderly participants enumerated, 1,182 (78.1%) were examined. After excluding 356 participants with VI in the better eye, data were analysed for the remaining 826 participants. The mean age (standard deviation) of these participants was 74.4 ± 8.4 years; 525 (63.6%) were women, and 111 (13.4%) had no schooling. The prevalence of unilateral VI was 38.1% (95% CI: 34.8–41.5; n = 315). Cataract (37.5%; n = 118) was the leading cause of UVI followed by Uncorrected Refractive Error (22.2%; n = 70) and posterior capsular opacification (18.4%; n = 58). The overall INDVFQ score was higher among those with UVI than those without UVI (37.7 versus 34.5; p < .01) suggestive of poor visual functioning.

**Conclusions:**

UVI was common and largely due to avoidable causes among the elderly in residential care with an adverse impact on visual functioning. Screening for vision loss in ‘homes for the aged’ and the provision of appropriate services should become a routine practice to achieve the goal of healthy aging in India.

## Introduction

India is home to over 275 million people with vision impairment (VI) including, over 137.2 million people with near VI.^1^ VI is associated with aging, with over 85% of those with VI reported to be 50 years or older age.^1^ By 2050, it is projected that every fifth Indian would be in the elderly age group (60 years and older).^2^ With an increasing proportion of elderly in the population, the number of people having VI is likely to increase in the future. Moreover, their living arrangements are also changing with the changes in society. The number of elderly either living alone or with their spouse or moving to a home for the aged has increased.^3^ A higher prevalence of vision loss has also been reported among the elderly living in residential care both in India and world over.^4–9^

Typically, VI is defined as the presenting or best-corrected visual acuity of 6/18 or worse in the better eye.^1^ This definition excludes people with unilateral VI (UVI). Several conditions such as corneal scars, posterior capsular opacification (PCO) after cataract surgery, and other conditions which often present as asymmetric are not included when better eye acuity is applied. Only a few population-based studies have reported the prevalence of UVI.^10–15^ In a few of these studies, the prevalence of UVI equals bilateral VI,^13,16^ while, UVI exceeds the prevalence of VI based on the better eye definition in other studies.^17,18^ While it is well-established that bilateral VI affects visual functioning and the quality of life,^19^ the impact of UVI on visual functioning is limited to few studies.^10,15^ These studies have reported on the negative impact of UVI on visual functioning.^10,15^ Delayed cataract surgery in the second eye has shown a negative impact on the individual’s quality of life.^16^ Improvement in quality of life and subjective visual functions such as binocular visual acuity and stereo acuity after second eye cataract surgery have also been reported.^20–22^ However, there are no studies reporting on UVI in the elderly age group in residential care in India.

The Hyderabad Ocular Morbidity in Elderly Study (HOMES) was designed to assess the prevalence, causes, risk factors, and impact of VI among the elderly in residential care facilities in the Hyderabad region in Telangana, India.^23^ Based on the visual acuity in the better eye, the prevalence of VI was 30.1%.^24^ We also reported the impact of bilateral VI on visual functioning in these participants.^19^ In this paper, we report on the prevalence, causes and impact of UVI on the visual functioning of the elderly living in residential care.

## Materials and methods

### Ethics approval

The Institutional Review Board of Hyderabad Eye Research Foundation, L V Prasad Eye Institute, approved the study protocol. Our study adhered to the tenets of the Declaration of Helsinki. All the participants were enrolled after providing written informed consent.

The sample size estimation for varying anticipated prevalence is reported in the previous publications.^23^ In short, each ‘home for the aged’ centre is considered as a cluster. Based on an anticipated prevalence of avoidable visual impairment of 20%, a precision in the estimate of the prevalence of 20%, a non-response rate of 25%, a design effect of 1.4 to account for clustering, a sample size of 666 individuals is required. The prevalence estimates were based our previous publication on VI in the elderly in residential care in a rural location.^4^ Anticipating a lower prevalence of 15% as the study is planned in an urban location, the final sample size selected 916 participants. However, all the homes in the Hyderabad region that have provided the consent for participation were included. Residents aged ≥60 years at the time of enumeration and residing in the home for the aged for at least one month were included in the study.^23,24^

After taking the informed consent of the participants, a detailed interview was conducted by the trained investigators. In addition to personal and demographic (age, gender, education level) information, it included information on other risk factors (smoking or alcohol consumption), self-reported systemic conditions (Diabetes, Hypertension) and mobility status (independently mobile, mobile with assistance or bedridden/ immobile).

The visual functioning was assessed using the validated Indian Vision Function Questionnaire (INDVFQ).^25,26^ This INDVFQ questionnaire was psychometrically validated for use in the elderly population in residential care.^27^ The INDVFQ has 33 questions in four domains (mobility, activity limitation, psychosocial impact and visual symptoms).^25,26^ Each question has four or five response options on difficulty or frequency using a Likert scaling from ‘no problem at all’ to ‘cannot do this because of vision’ for five response categories, and from ‘no problem at all’ to ‘cannot do’ for four response categories. Six questions were not applicable to the elderly in residential care as described in our previous publication.^19,27^ The final questionnaire had 27 questions.^19,27^ A higher score on the scale represents a higher degree of difficulty or a poorer function. The INDVFQ was not administered to the participants who were bedridden. In addition, the participants with Hindi Mini Mental State Examination (HMSE) score of less than 20 were also excluded as this score is suggestive of mild cognitive impairment. The questions in INDVFQ are related to memory and recall and hence mild cognitive impairment may affect their responses to the questionnaire.

### Eye examination

Trained examiners conducted the clinical assessments in ‘makeshift’ (temporary) clinics set up in each home for the aged. The clinical assessment protocol has been described in our previous publications.^23,24^ In brief, the clinical examination included visual acuity assessment, refraction, anterior and posterior segment examination. Distance and near visual acuity (VA) were assessed using a logMAR chart (logarithm of Minimum Angle of Resolution) at three metres and 40 centimetres, respectively. Tumbling E chart and English charts were used as needed. Also, presenting, pinhole, and best-corrected visual acuity were assessed. Anterior segment examination was done using a portable handheld slit lamp biomicroscope (BA 904 Haag-Streit Clement Clarke International, UK). Fundus images were taken using a non-mydriatic fundus camera (Zeiss Visuscout 100), and they were graded by trained graders. Participants having VI due to uncorrected refractive errors were provided with spectacles, and those who needed further care were referred to the L V Prasad Eye Institute for service provision. All eye care services and spectacles were provided at no cost to the participants.

### Definitions

Unilateral visual impairment (UVI) was defined as presenting VA worse than 6/18 in one eye and presenting VA 6/18 or better in the other eye. UVI was further classified as moderate UVI (presenting VA worse than 6/18 but better than or equal to 6/60), severe UVI (presenting VA worse than 6/60 to 3/60), and unilateral blindness (presenting VA worse than 3/60).^12,13^ Individuals with presenting VA worse than 6/18 in the better eye were not included in the analysis. The main cause of VI was assigned by the clinician for each eye as described in our previous publication.^23,24^ Wherever there were multiple causes from the clinical examination and the retinal images, the cause that was more likely to explain the vision loss was considered as the main cause of VI in that eye.^23,24^

### Data management

Data analysis was carried out using Stata Statistical Software Version 14.^28^ The participants with VI based on the better eye were excluded from analysis. Prevalence estimates were calculated and presented along with 95% CI. The association between personal and sociodemographic risk factors and UVI was assessed using multiple logistic regression analysis. The model fit was tested with Hosmer-Lemeshow goodness-of-fit test. Adjusted ORs were presented along with 95% CI. Statistical significance was assessed at the conventional level of p-value less than 0.05 (two-sided). However, exact p values were presented. The scores for each of the INDVFQ domains were calculated as the sum of the response scores divided by the maximum possible score and multiplied by 100 to get a domain score. Similarly, the overall INDVFQ score was calculated as the simple mean of the responses for each of the questions as reported in other studies that used this questionnaire.^26,29,30^ The independent sample t-test was used to compare mean INDVFQ scores of those with UVI and without UVI and reported with standard deviation.

## Results

### Study participants

Of the total 1,513 elderly participants enumerated, 1,182 (78.1%) were included in the study. After excluding 356 participants with VI in the better eye, data were analysed for the remaining 826 participants who were categorised as normal using VI definitions based on the better eye. The mean age (standard deviation) of these participants was 74.4 ± 8.4 years; 525 (63.6%) were women, and 111 (13.4%) had no schooling, 529 (64.0%) had school education and 186 (22.5%) had higher education. Over three-quarters of the participants (75.9%; n = 627) were widowed, separated or single. And the remaining 199 (24.1%) participants were married..

In total, 365 (44.2%) of the participants were from private/paid homes, 349 (42.3%) were from an aided or partially subsidized home, and the remaining 112 (13.6) were from free homes. Diabetes and hypertension were reported by 261 (31.6%) and 500 (60.5%) participants, respectively. In total, 530 (64.2%) participants were independently mobile, 243 (29.4%) needed assistance for mobility, and the remaining 53 (6.4%) participants were bedridden/immobile. Of the 826 participants, 104 (12.6%) had mobility and other health issues and 39 (4.7%) had low scores on HMSE. INDVFQ was administered on the remaining 683 participants (82.7%) who were eligible. The characteristics of the participants and the prevalence of UVI under each group are shown in Table 1.

### Prevalence of UVI

The prevalence of UVI was 38.1% (95% CI: 34.8–41.5; n = 315). This included 221 (26.8%) participants with moderate VI (95% CI: 23.8–29.9), 22 (2.7%) participants with Severe VI (95% CI: 1.7–4.0), and 72 (8.7%) participants with blindness (95% CI: 6.9–10.6).

### Causes of UVI

**Figure 1:**
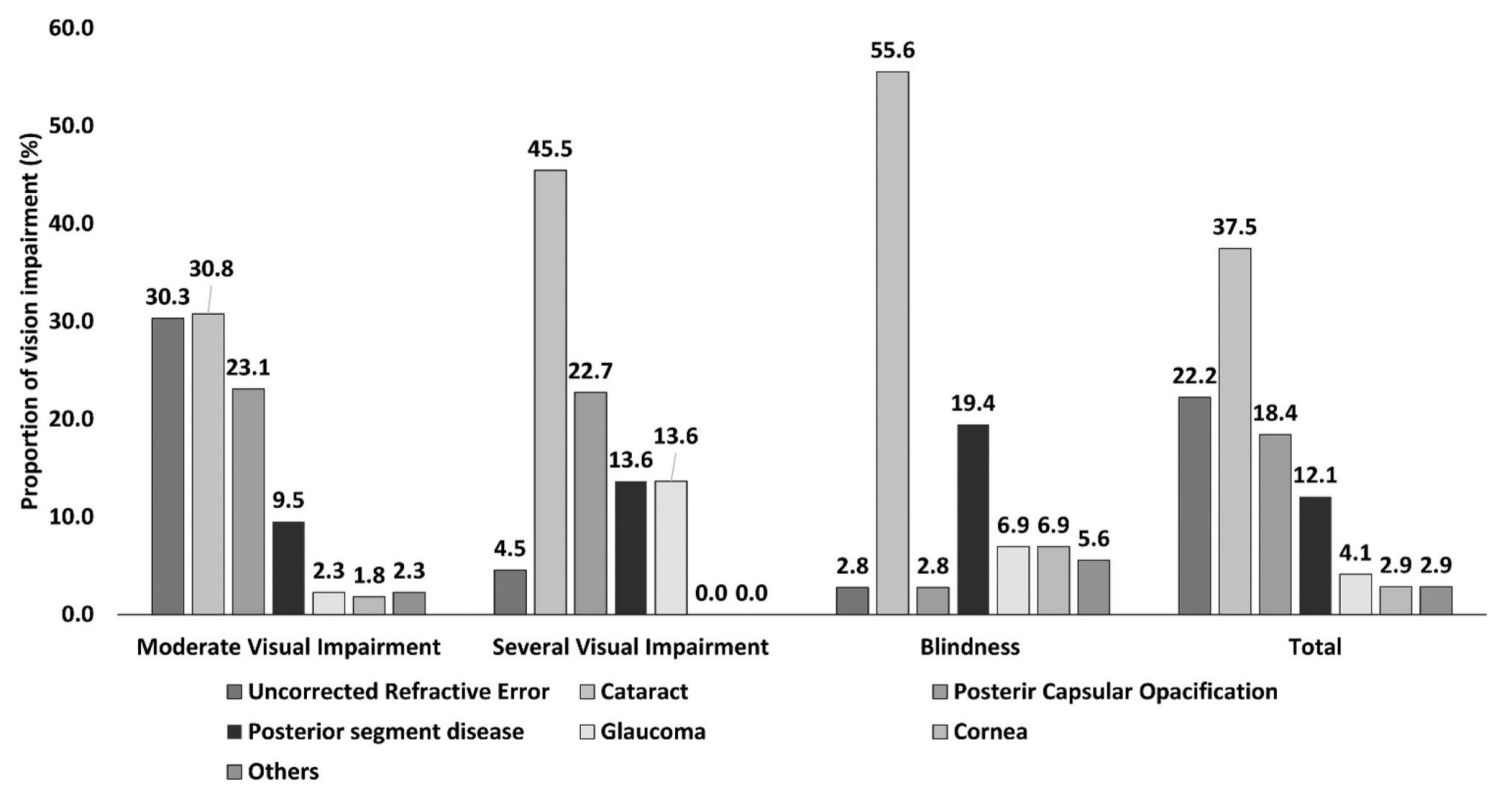
Distribution of the causes of vision impairment (n = 315) among the elderly living in a Home for the Aged.

Cataract (37.5%; n = 118) was the leading cause of UVI followed by Uncorrected Refractive Error (22.2%; n = 70) and PCO (18.4%; n = 58). The other causes for UVI were posterior segment disease (12.1%, n = 38), glaucoma (4.1%, n = 13) or corneal disease (2.9%, n = 9) and other ocular diseases (2.9%, n = 9). Overall, 78.1% (n = 246) of UVI was either treatable or correctable. The causes of UVI stratified by categories of VI are shown in Figure 1. Cataract was the leading cause of blindness, and severe and moderate UVI. PCO was the second leading causes of severe and moderate UVI, and posterior segment disease is the second leading cause of blindness.

### Risk factors for UVI

Both simple regression analysis and multiple logistic regression showed that being in the older age group (80 years or older), lower levels of education, living in free homes and those who were immobile/bedridden had higher odds for UVI. On multiple logistic regression analysis, the elderly in the 80 years and older age group were twice likely to have UVI (OR:1.87;95% CI: 1.24–2.81). Participants who had some formal schooling (OR: 1.70; 95% CI: 1.13–2.56) were nearly twice as likely to have UVI than those with higher education. Also, participants who had no education (OR: 2.62; 95% CI: 1.49–4.59) were nearly three times more likely to have UVI than those with higher levels of education. Compared to the elderly living in a private home, those in a free home were nearly twice as likely to have UVI (OR: 1.88; 95% CI: 1.16–3.04). Similarly, compared to the elderly participants who were independently mobile, those who were immobile or bedridden were more than twice as likely to have UVI (OR: 2.28; 95% CI: 1.25–4.16). Gender, marital status, diabetes, hypertension, smoking, and alcohol consumption were not associated with UVI. (Table 2)

### Impact of UVI on visual functions

Participants with UVI had significantly higher scores suggesting a poorer visual functioning in all domains of the INDVFQ except the visual symptom domain. The higher difference in visual function scores of participants with UVI than those without UVI was noted for the psychosocial domain (14.1%), mobility scores (10.9%) and activity limitation scores (10.1%). The overall INDVFQ score was higher among those with UVI than those without UVI (37.7 versus 34.5; p < .01) suggestive of poor visual functioning. (Table 3)

## Discussion

Over 38% of the elderly in residential care had UVI in this study. UVI was more prevalent than VI based on better eye definition. In summary, overall, two-thirds of the elderly had either UVI or bilateral VI as reported in the earlier publication.^24^ This is cause of concern as both bilateral VI and UVI have an adverse impact on the visual functioning of the elderly in residential care.^19,27^

Similar to that of bilateral VI, a large proportion of UVI could be either corrected with spectacles (refractive error) or treated with surgery (cataract) or laser capsulotomy (PCO).^24^ The emergence of PCO as an important cause of UVI in the elderly has not been reported earlier. This finding has implications for service provision and indicates that cataract can no longer be considered a one-time intervention. As reported in our previous publication, given the increasing cataract surgical rate in India alongside increasing life expectancy, there is also the likelihood that PCO will increasingly be one of the most important causes of UVI.^24^

UVI was associated with the older age group, poor mobility, lower level of education, or residing in a free homes /non-paying for the aged centres. Elderly persons with UVI may tend to play down their vision loss, as they function without much effort binocularly in a real-world situation. However, there is no evidence to support this assumption. It could be particularly true for those in the oldest age group as we found higher odds for UVI after adjusting for other covariates. It is also possible that other health issues took precedence over poor vision in one eye in older age groups. For instance, the limited options for an active lifestyle or access to resources may preclude the uptake of eye care services. It may thus result in a higher prevalence of UVI among those living in the Homes for the aged facilities. The prevalence of UVI was lower in community-dwelling elderly in the community-based study from the same state (34.5% versus 38.0%).^14^

Similar to the association between level of education and bilateral VI, UVI was higher among those with lower levels of education. A lower level of education may serve as a surrogate indicator for lower socioeconomic status and fewer resources to seek eye care. This corroborates well with higher odds of UVI that was noted among those living in free homes compared to paying or subsidized homes. Another population study on elderly in the same region also reported a higher prevalence of bilateral VI among those without education.^31^ Inability to afford a pair of spectacles or cataract surgery could prevent elderly residents from seeking eye care. In addition, poor mobility leads to loss of independence, making the individual dependent on either their kin or home authorities to escort them to an eye care service provider. Also, they may have less visual demand and not feel any need for seeking eye care services unless the vision in their better eye is very poor. There are also reports on the poor uptake of services after being referred among the residents of nursing homes because one or more family members are unwilling to accompany them for eye care.^32^ Lack of a felt need was also reported as a major reason for not utilizing the services among community-dwelling individuals with UVI, as has been quoted in earlier studies from this area.^12^ Lack of felt need was also reported as the leading reason for poor uptake of services among the elderly both in residential care and in the population in the region.^33,34^

UVI significantly impacts the visual functioning of the elderly. Earlier studies reported on the adverse impact of bilateral vision loss on visual functioning and quality of life among the elderly in the general population.^29,30^ But there are only a few studies reporting on the elderly in residential care.^19,35^ Researchers from Australia reported an adverse impact on visual functioning among those with UVI, though the impact was less when compared to those with bilateral vision loss.^10,15^ As UVI is based on worse eye acuity, we did not find a significant difference in the visual symptom domain. Significantly, all other domains were affected among those with UVI. The impact was highest in the psychosocial domain which may be due to an inherent fear of loss of independence due to increased vision loss in the other eye.

This is the first study to report on UVI and its impact on the visual functioning of the elderly in residential care in India. While earlier studies have established that there is a 30% prevalence of VI based on better eye acuity, our report uncovered that as much as 38% of the elderly had UVI. It translates to over two-thirds of the elderly having either UVI or VI based on the better eye. Thus, aggressive strategies are warranted to address vision loss in the elderly, targeting service delivery for those living in a home for the aged in India. A holistic intervention involving correction of refractive errors, provision of good quality cataract surgery, and ophthalmic lasers for PCO need to be implemented. The data on bilateral VI and UVI can help plan eye care services appropriately.

The strengths of this study are that it included a large sample of the elderly in residential care with a good response rate, and a comprehensive eye examination was done for all participants. There is growing evidence suggesting a link between VI, visual functioning and cognition.^36–38^ We had excluded the participants with cognitive impairment as the questionnaire that was used required good memory and recall for consistent responses. Future studies may include more robust tools for cognition and objective assessment of visual functioning in this population. As the current study was done among the residents in homes for the aged centres, the results may not be extrapolated to the general population.

In conclusion, UVI is of great concern among the elderly in residential care, adversely affecting their visual function. As a large proportion of UVI can be addressed and managed, formulating guidelines for assessment and designing effective strategies to provide eye care in homes for the aged will result in better visual functions and better well-being for the elderly in India.

## Figures and Tables

**Table 1:** The characteristics of participants (n = 826) examined in the HOMES study.

	Total	Unilateral VI (n)	Unilateral VI (%)	p-Value
**Age group (years)**				0.002
60–69	244	80	32.8	
70–79	330	117	35.5	
80 and above	252	118	46.8	
**Gender**				0.476
Male	301	110	36.5	
Female	525	205	39.0	
**Marital status**				0.515
Married	199	71	35.7	
Widowed/separated/	627	243	38.8	
single				
**Education level**				<0.01
No Schooling	111	58	52.3	
School education	529	207	39.1	
Higher education	186	50	26.9	
**Hypertension**				0.354
Yes	500	197	39.4	
No	326	118	36.2	
**Diabetes**				0.076
Yes	261	88	33.7	
No	565	227	40.2	
**Mobility score**				0.002
Immobile/Bedridden	53	30	56.6	
Mobile with support	243	102	42.0	
Independently mobile	530	183	34.5	
**Type of home**				0.013
Private home	365	126	34.5	
Aided/Partially paid	349	133	38.1	
Free	112	56	50.0	
**Smoking status**				0.771
Never	683	262	38.4	
Current/past	143	53	37.1	
**Alcohol consumption**				0.414
Never	689	267	38.8	
Current/past	137	48	35.0	
**Total**	**826**	**315**	38.1	

**Table 2:** Association of unilateral visual impairment with sociodemographic characteristics, systemic conditions and personal history identified using multiple logistic regression analysis (n = 826).

	Crude Odds Ratio (95% CI)	p value	Adjusted Odds Ratio (95% CI) †	p value
**Age group (years)**				
60–69	Reference		Reference	
70–79	1.23 (0.79–1.60)	0.51	1.16 (0.80–1.68)	0.437
80 and above	1.81 (1.25–2.60)	<0.01	1.87 (1.24–2.81)	0.003
**Gender**				
Male	Reference		Reference	
Female	1.11 (0.83–1.49)	0.476	0.85 (0.56–1.30)	0.464
**Education level**				
Higher education	Reference		Reference	
School education	1.75 (1.21–2.53)	<0.01	1.70 (1.13–2.56)	0.011
No education	2.98 (1.82–4.88)	<0.01	2.62 (1.49–4.59)	0.001
**Marital status**				
Married	Reference		Reference	
Widowed/ separated/ single	0.90 (0.64–1.23)	0.515	0.99 (0.69–1.41)	0.944
**Home type**				
Private	Reference		Reference	
Aided/Partially paid	1.17 (0.86 01.59)	0.32	1.19 (0.85–1.66)	0.30
Free/Non-paying	1.90 (1.24–2.91)	<0.01	1.88 (1.16–3.04)	0.01
**Diabetes**				
No	Reference		Reference	
Yes	0.76 (0.56–1.03)	0.08	0.84 (0.60–1.17)	0.301
**Hypertension**				
No	Reference		Reference	
Yes	1.15 (0.86–1.53)	0.354	1.07 (0.79–1.47)	0.654
**Smoking status**				
Never	Reference		Reference	
Current/past	0.95 (0.65–1.37)	0.77	1.13 (0.68–1.90)	0.634
**Alcohol consumption**				
Never	Reference		Reference	
Current/past	0.85 (0.58–1.25)	0.414	0.85 (0.53–1.37)	0.512
**Mobility score**				
Independently mobile	Reference		Reference	
Mobile with support	1.37 (1.00–1.87)	0.047	1.20 (0.85–1.68)	0.299
Immobile/bedridden	2.47 (1.40–4.38)	<0.01	2.28 (1.25–4.16)	0.007

† Hosmer-Lemeshow Goodness of fit test for the regression model, p = 0.87

**Table 3:** Comparison of Indian Visual Function (INDVFQ) scores among participants with or without Unilateral Visual Impairment (UVI).

Domain of INDVFQ	Mean (SD)† Unilateral VI (n = 230)	Mean (SD)† No Unilateral VI (n = 453)	Mean difference (% difference)	p value
Mobility	26.4 (12.2)	23.8 (8.9)	2.6 (10.9)	<0.01
Activity limitation	28.1 (10.9)	25.5 (8.2)	2.6 (10.1)	<0.01
Psychosocial impact	34.5 (16.6)	30.2 (11.3)	4.3 (14.1)	<0.01
Visual symptoms	39.8 (14.1)	37.5 (13.8)	2.3 (6.1)	0.041
**Overall INDVFQ score**	37.7 (13.2)	34.5 (10.5)	3.2 (9.3)	<0.01

† Higher score denotes poorer visual functioning
